# Abnormal Localization of STK17A in Bile Canaliculi in Liver Allografts: An Early Sign of Chronic Rejection

**DOI:** 10.1371/journal.pone.0136381

**Published:** 2015-08-25

**Authors:** Munetaka Ozeki, Adeeb Salah, Wulamujiang Aini, Keiji Tamaki, Hironori Haga, Aya Miyagawa-Hayashino

**Affiliations:** 1 Department of Forensic Medicine, Graduate school of Medicine, Kyoto University, Kyoto, Japan; 2 Department of Diagnostic Pathology, Kyoto University Hospital, Kyoto, Japan; 3 Experimental and Clinical Research Center, Diabetes and Research Laboratory, Kocaeli University, Izmit, Turkey; 4 Center for Innovation in Immunoregulative Technology and Therapeutics, Graduate School of Medicine, Kyoto University, Kyoto, Japan; Texas A&M Health Science Center, UNITED STATES

## Abstract

The biological significance of STK17A, a serine/threonine kinase, in the liver is not known. We analyzed STK17A expression in HepG2 cells and human liver tissue. Accordingly, we investigated whether STK17A could help in identifying earlier changes during the evolution of chronic rejection (CR) after liver transplantation. RT-PCR and immunofluorescence were used to analyze STK17A expression in HepG2 cells. Antibody microarray was performed using human liver samples from CR and healthy donors. Immunohistochemistry was used to verify the clinical utility of STK17A on sequential biopsies for the subsequent development of CR. A novel short isoform of *STK17A* was found in HepG2 cells. STK17A was localized in the nuclei and bile canaliculi in HepG2 cells and human livers. Microarray of STK17A revealed its decrease in failed liver allografts by CR. During the evolution of CR, the staining pattern of bile canalicular STK17A gradually changed from diffuse linear to focal intermittent. The focal intermittent staining pattern was observed before the definite diagnosis of CR. In conclusion, the present study was the first to find localization of STK17A in normal bile canaliculi. Abnormal expression and localization of STK17A were associated with CR of liver allografts since the early stage of the rejection process.

## Introduction

Serine/threonine kinase 17A (STK17A), also known as death-associated protein kinase-related apoptosis-inducing protein kinase 1 (DRAK1), is a member of the death-associated protein kinase (DAPK) family [1–4]. Originally, *STK17A* was cloned from a human placenta cDNA library [2]. While its catalytic domain is related to that of DAPK, STK17A lacks a death domain and a Ca^2+^/calmodulin regulatory domain [2]. *STK17A* mRNA expression in the liver has been shown by Northern blotting analysis, although the expression was seen to be relatively lower than in other tissues, and its biological significance in the liver has not been studied [2]. We recently found that STK17A was expressed in the human hepatocellular carcinoma cell line HepG2, forming bile canaliculi *in vitro*. An association has been reported between bile canaliculi network formation/maintenance and kinases, such as liver kinase B1 (LKB1) and adenosine monophosphate (AMP)-activated protein kinase (AMPK) [5,6]. A recent report has indicated that DRAK in *Drosophila* regulates the actin dynamic *in vivo* and plays a role in signaling networks that shape epithelial tissue [7]. Given the role of DRAK in cell structure as well as its localization, we investigated whether STK17A could clarify the bile canalicular changes during the evolution of chronic rejection (CR) after liver transplantation.

The recognition and control of liver allograft rejection has undergone clear improvement; nevertheless, CR remains a serious cause of allograft dysfunction and consequent graft loss [8–12]. This is partly due to the classical presentation of graft dysfunction, which occurs less commonly during the first post-transplant year, with more cases having an indolent but progressive course over a period of several years before manifestation of CR [8–12]. There is no specific anti-CR drug; therefore, re-transplantation is the standard treatment for advanced cases [9]. The mechanisms of CR are still incompletely understood and the etiology is multifactorial [9]. Many factors exacerbate CR, such as acute or recurrent rejection [8,10,11,12], viral infection [13], antiviral therapy [14,15], and low levels of immunosuppression [13,15]. Currently, CR is thought to be caused by cellular as well as humoral immunity [9–11,16,17].

The diagnostic features of CR in liver allografts include obliterative lesions in medium-sized and large arteries, which are rarely seen in needle biopsies, and loss of interlobular bile ducts from >50% of portal areas [9,10]. Bile duct loss in >50% of portal areas represents a late stage of CR and is likely to be irreversible [9,10]. However, symptoms may be reversed if the bile duct loss is limited to <50% of the portal areas [9,18]. The early, reversible stage of CR is characterized by inflammatory and degenerative changes of the biliary epithelium, even before detection of evident bile duct loss [9,10,19]. Since loss of bile ducts in <50% of portal areas or degenerative changes of bile ducts are also seen in other conditions, including bile duct stenosis or duct damage due to hepatic artery thrombosis, it is important to have a consistent clinical picture and to recognize the histological features of CR in order to make a diagnosis of CR [10]. Therefore, biomarkers closely associated with CR would be helpful for understanding the mechanism, as well as for identifying early changes in the progression of CR.

In the current study employing HepG2 cells, we found a new location for STK17A in bile canaliculi. Accordingly, using antibody microarray and immunohistochemistry, we provide evidence about alteration of STK17A expression during CR after liver transplantation. Our findings indicate that alteration in the expression pattern of STK17A in bile canaliculi during the progression of CR may help identify early stages of the disease.

## Materials and Methods

### cDNA sequence

HepG2 cells were provided by the RIKEN BioResource Center (Tsukuba, Ibaraki, Japan) through the National Bio-Resource Project of the Ministry of Education, Culture, Sports, Science and Technology (MEXT), Japan. The cells were maintained in minimum essential medium (Life Technologies, Carlsbad, CA, USA) supplemented with 10% fetal bovine serum (Thermo Fisher Scientific Inc., Waltham, MA, USA) and antibiotics/antimycotics (Life Technologies) at 37°C in a 5% CO_2_ incubator.

Total RNA from the HepG2 cells was prepared using TRIzol (Life Technologies) according to the manufacturer's method with subsequent RQ1 RNase-free DNase treatment (Promega, Madison, WI, USA) and transcribed into cDNA with the SuperScript III cDNA Synthesis Kit (Life Technologies). A *STK17A* cDNA fragment was amplified in the reaction mixture containing Promega GoTaq Master Mix by PCR with 30 cycles of denaturation at 95°C for 45 seconds, annealing at 59°C for 45 seconds, and extension at 72°C for 1 minute. The following primer set was used: GAACACCATGATCCCTTTGG and GTGCCTTTTCCATCCTGAAA. The cDNA fragment separated in agarose gel was recovered and cloned using the pGEMT Easy Vector System (Promega). A DNA sequence reaction was carried out using the BigDye Terminator ver 3.1 Cycle Sequencing Kit (Life Technologies), and the resulted fragments were analyzed on the 310 Genetic Analyzer (Life Technologies). The prediction of alternative splicing sites was carried out by the Alternative Splice Site Predictor (http://wangcomputing.com/assp/index.html) [20].

### Immunofluorescence imaging

HepG2 cells cultured on cover-chamber (Matsunami Glass Ind., Ltd., Osaka, Japan) were fixed with 4% paraformaldehyde in phosphate buffer and permeabilized with 0.01% Triton X-100 in phosphate buffered saline. Fixed cells were stained with anti-human STK17A monoclonal antibody (Abcam, Cambridge, UK) and detected with Alexa Fluor 488-labeled anti-mouse IgG (Life Technologies). F-actin staining was carried out with Alexa Fluor 594 phalloidin (Life Technologies). The nuclei were visualized with DAPI. Fluorescence imaging analysis using the Leica TCS SP8 laser scanning microscope (Leica Microsystems GmbH, Wetzlar, Germany) with photon counting mode was performed at the Medical Research Support Center, Graduate School of Medicine, Kyoto University, which was supported by the Platform for Drug Discovery, Informatics, and Structural Life Science from the MEXT, Japan.

### Protein microarray analysis

Protein was extracted from frozen liver tissue, and candidate marker proteins for CR diagnosis were identified by using the Panorama Antibody Microarray XPRESS Profiler 725 Kit (Sigma-Aldrich, Tokyo, Japan). The GenePix 4400A scanner (Molecular Devices, Tokyo, Japan) and Array-Pro Analyzer ver. 4.5 (Media Cybernetics, Nippon Roper, K.K., Tokyo, Japan) were used for quantile normalization of samples to obtain a protein level expression file. Unpaired Student’s *t*-test was performed to identify the significantly (P<0.05) increased or decreased expression between donor livers (N = 20) and failed allografts due to CR (N = 5).

### Western blot analysis

Frozen liver tissue specimens from failed liver allografts due to CR and healthy donor livers were homogenized in lysis buffer (20 mM Tris, pH 7.4, 2 mM EDTA, 150 mM NaCl, 0.5% Triton X-100, and 1 μl of protease inhibitor cocktail). Samples were centrifuged and supernatants collected for total protein content analysis by the bicinchoninic acid method (BCA Protein Assay Kit; Thermo Fisher Scientific, Inc.). Equal amounts of protein extract from each sample were separated by electrophoresis on 10% sodium dodecyl sulfate-polyacrylamide gels and then transferred to polyvinylidene difluoride membrane (BIO-RAD, Hercules, CA, USA). Blots were blocked at room temperature (RT) for 10 minutes in 5% skim milk in phosphate buffered-saline containing 0.05% Tween 20 (PBST) containing 0.05% Tween 20 and then incubated overnight at RT in primary antibody (rabbit polyclonal anti-human STK17A; Sigma-Aldrich, St-Louis, MO, USA). The membrane was washed three times with PBST and incubated at RT for 180 minutes with secondary antibody (goat anti-rabbit IgG- HRP, Santa Cruz Biotechnology, Dallas, TX, USA). The protein bands were visualized with a chemiluminescence substrate (Thermo Fisher Scientific, Inc.), and images were obtained using Ez-Capture MG (Daihan Scientific Co., Ltd., Gangwon-do, South Korea). Membrane was stripped in 0.2M NaOH and reprobed with monoclonal anti-beta-actin antibody (mouse monoclonal, Abcam). Visualized bands were analyzed using the CS Analyzer (Atto Corporation, Tokyo, Japan).

### Human liver tissue and immunohistochemistry

Human liver tissue was harvested at re-transplantation from 5 failed liver allografts due to CR, and from sequential biopsies from 5 pediatric liver transplant recipients with clinically and histologically established CR. For sequential biopsy specimens, those taken before, at the time of, and after the diagnosis of CR were included. Healthy donor livers in each case that were taken before liver transplantation were also examined (N = 10). The cases and data were collected from the archive of the pathology department of Kyoto University Hospital. The specimens were selected from those obtained between 2006 to 2014.

Liver biopsy specimens or explanted liver tissue from re-transplantation were fixed in 10% buffered formalin. The paraffin-embedded specimens were sliced 3 μm thick, and stained either with H&E, Masson’s trichrome, or cytokeratin 7 (clone OV-TL 12/30, DakoCytomation, Glostrup, Denmark; 1:200 dilution). Immunohistochemical findings were assessed together with allograft histology and liver function tests. Rejection was diagnosed according to the Banff criteria [2]. The liver specimens were stained with STK17A antibody (anti-DRAK1 antibody, rabbit polyclonal, Sigma-Aldrich, St. Louis, MO, USA, 1:100 dilution). The well-characterized bile canalicular marker, bile salt export pump (BSEP) antibody (Clone F-6, mouse monoclonal, Santa Cruz Biotechnology, 1:100 dilution), was added for comparison. The REAL EnVision/HRP detection system (DakoCytomation) was used to detect immunohistochemical signals. In addition, a series of 9 biopsy specimens from 3 pediatric patients who had acute cellular rejection (ACR) episodes that subsequently resolved was also examined. The liver allograft biopsies were performed according to protocol or to determine the cause of allograft dysfunction. All sample collection and use of clinical records were performed under the written consent of patients and/or their families, and the study was conducted according to the principles expressed in the Declaration of Helsinki. The Ethics Committee of Kyoto University approved this study (E2374).

### STK17A immunohistochemical staining evaluation

STK17A staining was considered positive when linear continuous or discontinuous staining of bile canaliculi was identified with a 40 × objective lens. Then, a semiquantitative approach was applied; staining of >50% of the area of the needle biopsy core was defined as diffuse, while staining of <50% was defined as focal. Liver sections from healthy liver donors served as positive controls. A negative control consisted of replacing the anti-STK17A antibodies with an equivalent concentration of normal rabbit serum.

### Bioinformatic analysis of STK17A

The nuclear localization signal in STK17A was searched by a cNLS mapper [21]. The protein structure of STK17A was predicted by I-TASSER [22]. Amino acid alignment of the C-terminal region of STK17A with bile canalicular proteins (MPR2 and BSEP) was conducted by MAFFT version 7 [23].

## Results

### A novel short isoform of *STK17A* in HepG2 cells

**Fig 1:**
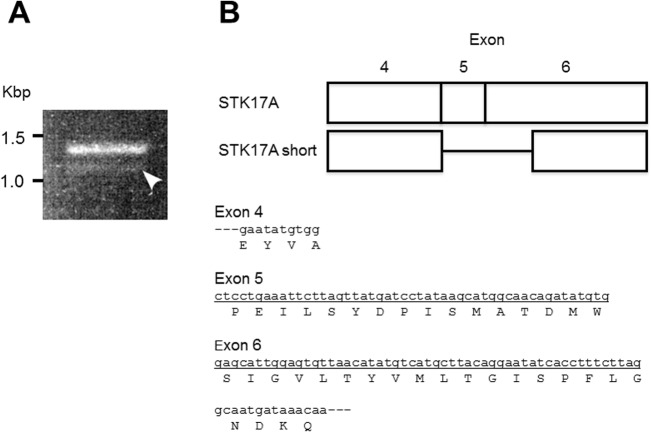
A short isoform of *STK17A* in HepG2 cells. (A) Polymerase chain reaction (PCR) of STK17A in HepG2 cells. The white arrowhead shows the band of the short isoform. (B) Schematic cDNA structure of a novel isoform of *STK17A*. The underlying cDNA sequence is lacking in the *STK17A* short isoform.

The *STK17A* gene is mapped on chromosome 7 and is composed of 7 exons. Primers for reverse transcription-polymerase chain reaction (RT-PCR) were designed to cover all exons. The forward primer locates on Exon 1 and the reverse primer on Exon 7. In analyzing the expression of *STK17A* mRNA in HepG2 cells, a novel short isoform was detected by RT-PCR unpredictably. We observed an approximately 100-bp smaller band compared to the full-length *STK17A* fragment, although its expression level was much lower (Fig 1A). The DNA band was excised from the gel and cloned into a vector for the sequence analysis. The result revealed that this band was a novel short isoform of *STK17A* lacking 102 nucleotides comprised of whole Exon 5 and a part of Exon 6, estimated to lack 34 amino acids, as shown in Fig 1B. The end of the skipped sequence was AG, a well-characterized acceptor site for alternative splicing [24]. Indeed, a computational prediction of *STK17A* alternative splicing showed that the position at 945 bp in the full-length mRNA sequence (cctttcttagGCAATGATAA, GenBank: 109255144) was an acceptor site [20].

**Fig 2:**
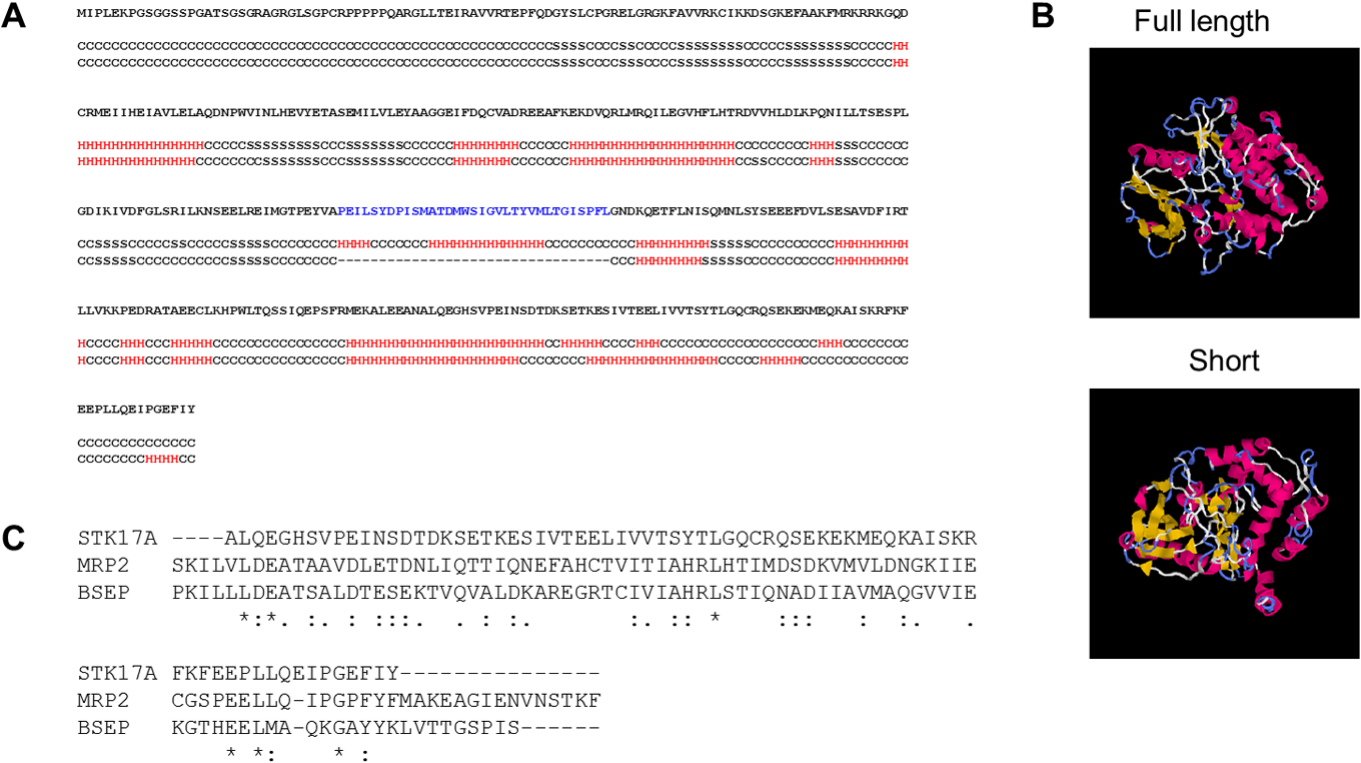
Structure of the short isoform of STK17A. (A) Secondary structure of the short isoform as predicted by I-TASSER. (B) 3D structure of the short isoform as predicted by I-TASSER. (C) Amino acid alignment between STK17A and well-characterized bile canalicular proteins shown by MAFFT version 7.

The short isoform still seems able to localize at the nucleus due to the classical monopartite nuclear localization signal (NLS) at amino acid position 91-FMRKRRKGQD by the cNLS mapper [21]. Moreover, the short isoform was predicted to have a different structure in 2D and 3D; that is, the secondary structure prediction by I-TASSER [22] showed a structural difference at the C-terminal region (Fig 2A). Although it was not in the kinase domain region, it can nevertheless cause conformational change in the 3D structure (Fig 2B). Then, we focused on the C-terminal region. The amino acid alignment study between STK17A and the well-characterized bile canalicular proteins MRP2 and BSEP using MAFFT version 7 [23] showed weak sequence similarity (Fig 2C). Although no bile canaliculi localization signal sequence has been reported, this region can be a possible site for such localization.

### STK17A localization in the liver

**Fig 3:**
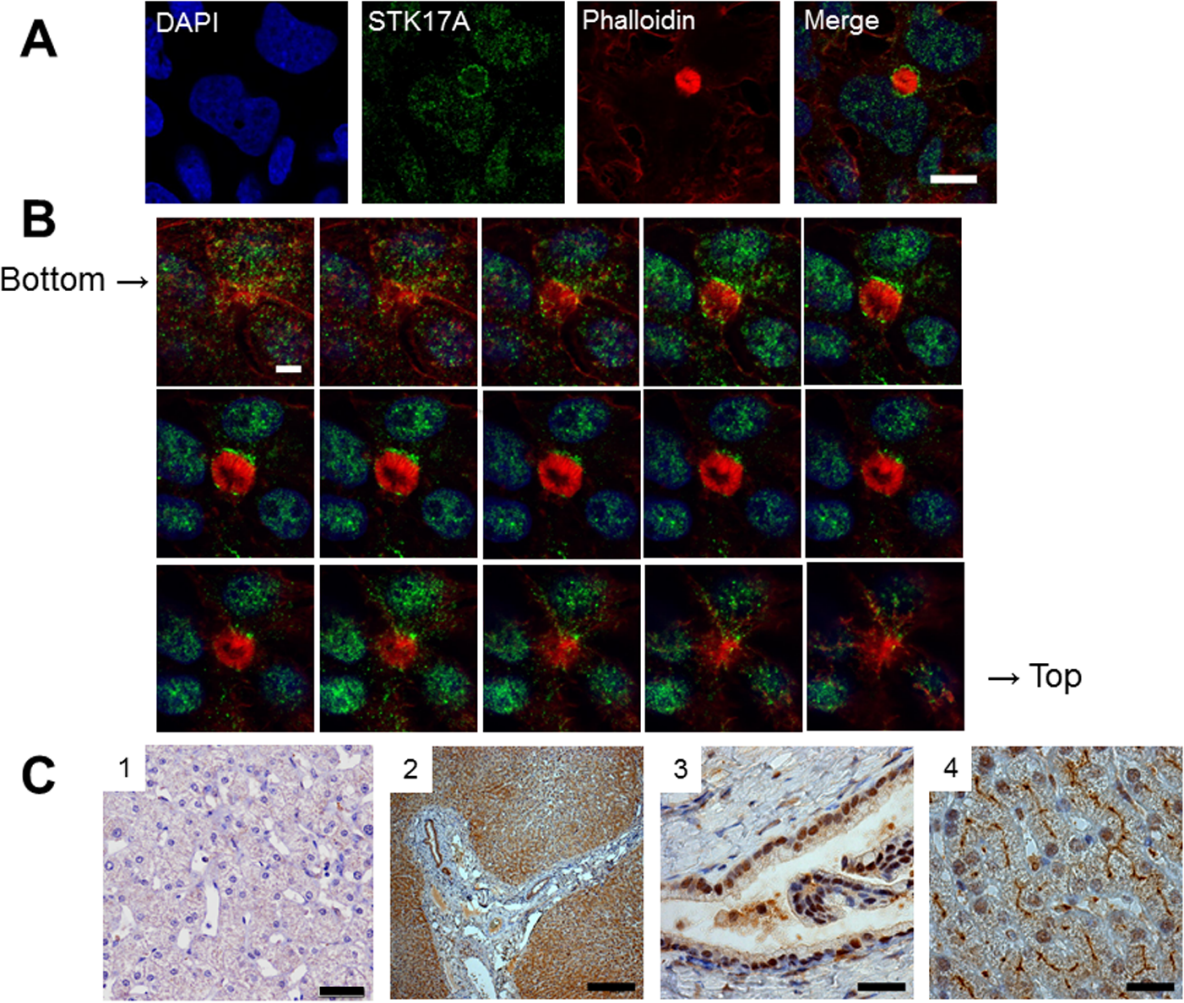
Bile canalicular localization of STK17A in HepG2 cells and human liver. (A) HepG2 cells are stained with anti-STK17A antibody (green). DAPI (blue) and phalloidin (red) are used as counterstaining for nuclei and F-actin, respectively. Fluorescence images are captured using laser scanning confocal microscopy. The arrowhead shows STK17A surrounding bile canaliculi in HepG2 cells. Scale bar: 10 μm. (B) Z-stack series of bile canalicular STK17A in HepG2. Scale bar: 5 μm. (C) Formalin-fixed paraffin-embedded human liver section stained with anti-STK17A antibody. (1) Negative control. Scale bar: 20 μm. (2) Low-power view of STK17A staining at the porta hepatis and hepatic lobules. Scale bar: 250 μm. (3) At the porta hepatis, bile duct epithelial cells have positive signals in the nuclei but not in the apical side. Scale bar: 25 μm. (4) Hepatocyte nuclei and bile canaliculi are positive for STK17A in the hepatic lobules. Scale bar: 25 μm.

**Fig 4:**
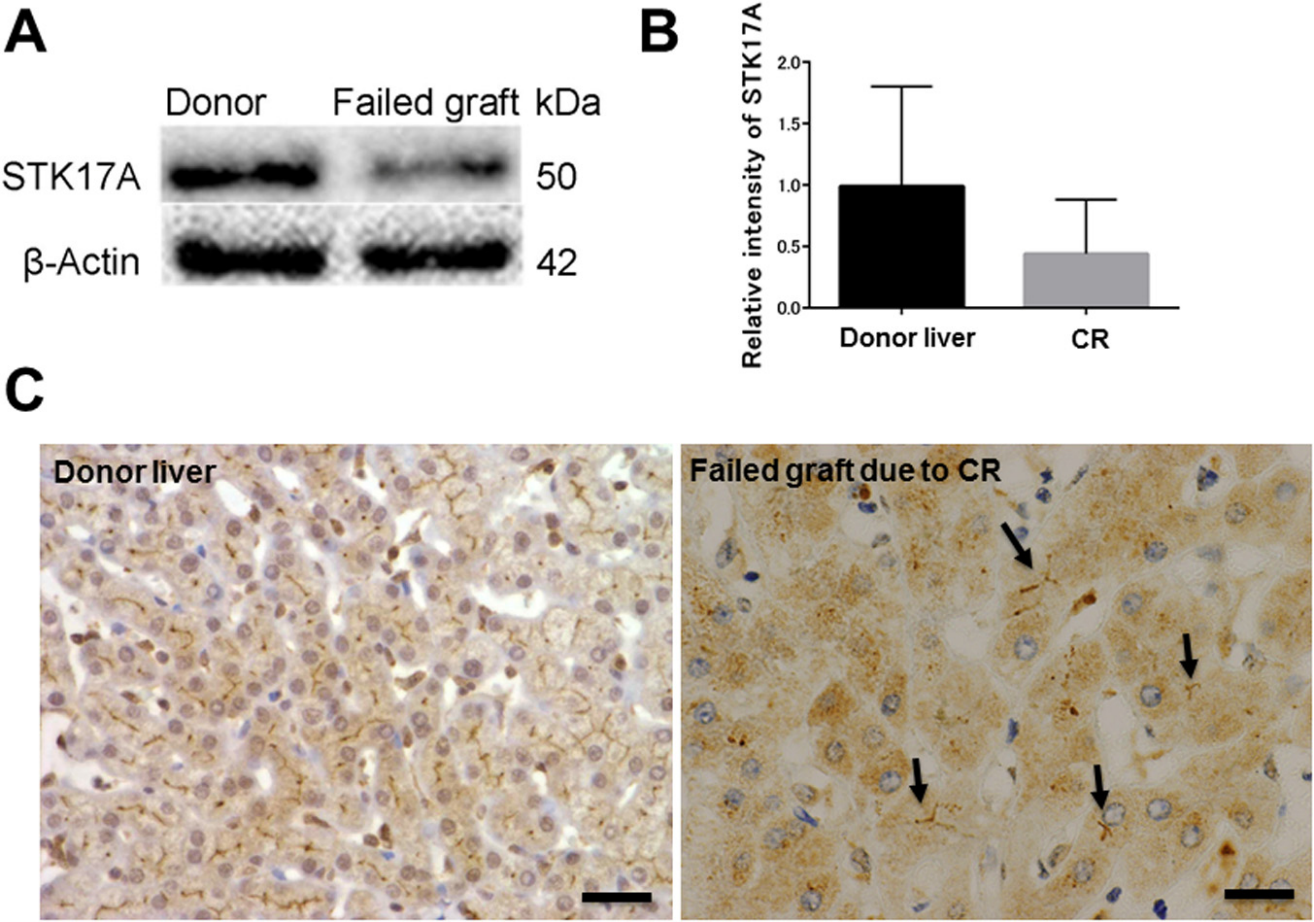
Confirmation of STK17A down-expression in failed allografts due to chronic rejection (CR). (A) Representative Western blot of a healthy donor liver and a failed liver allograft due to CR. This confirms the finding from the antibody microarray analysis that *STK17A* is downregulated in failed liver allografts due to CR. (B) Relative intensity level of STK17A normalized to that of beta-actin. The means of three independent experiments of normal liver samples and failed allografts due to CR (n = 3, each) were compared. Each bar represents mean ± SEM. (C) Immunohistochemistry for anti-STK17A antibody. Focal bile canalicular staining of failed allograft due to CR in contrast to healthy donor liver, which shows diffuse, linear staining. Arrows represent bile canaliculi. Scale bar: 20 μm.

As HepG2 cells are known to form bile canaliculi in two dimensional culture [25], we investigated the distribution of STK17A in HepG2 cells by immunofluorescence staining. STK17A was detected in the nuclei and slightly in the cytosol, as seen in Fig 3A. The lumens of the bile canaliculi were filled with microvilli. The bile canaliculi were visualized by fluorescence-labeled phalloidin, an F-actin binding peptide from the *Amanita phalloides* mushroom. STK17A was found to surround bile canaliculi in HepG2 cells (Fig 3A). In high-power viewing, STK17A accumulated around the bile canaliculi more heavily than around the nuclei. Confocal Z-stack images were collected at 0.29-μm intervals. Z-stack images of HepG2 cells showed staining at the nuclei as well as the apical membrane of the bile canaliculi. Bile canalicular networks were seen at the bottom and top images (Fig 3B). Immunostaining of liver sections from healthy donors with anti-STK17A antibody clearly visualized the nuclei and bile canaliculi (Fig 3C-2 and 3C-4). In contrast, the bile duct epithelial cells in the porta hepatis had positive signals only in the nuclei, but not in the apical side. This is the first evidence that STK17A localizes selectively in bile canaliculi in the liver. The negative control study demonstrated an appropriate staining result (Fig 3C-1).

### STK17A down-expressed in failed liver allografts due to CR

We employed antibody microarray technology to identify biomarkers for early detection of CR after liver transplantation. Liver samples from 20 healthy donors (median age 33, range 20~66 years) and 5 failed allografts due to CR (median age 25 years at the time of re-transplantation, range 18~38 years) were utilized as a discovery set for antibody microarray. Eight proteins were recognized, 5 were 2-fold down-expressed and 3 were 2-fold high-expressed in failed allografts due to CR compared with donor livers (Table 1). Remarkably, STK17A expression was 2-fold down-expressed in failed allografts due to CR. This reduction was confirmed by Western blot analysis, its densitometric level of STK17A (Fig 4A and 4B), and immunohistochemistry (Fig 4C). The STK17A band intensity was weaker in the failed liver allografts due to CR than in the donor liver samples. STK17A staining in the failed allografts was rare, and bile canaliculi were more severely destroyed than in normal livers.

**Table 1:** List of candidate proteins with 2-fold difference between donor livers and failed grafts due to CR by antibody microarray.

Protein	Fold change (CR/donor)
BID	0.356
STK17A	0.376
PUMA bbc3	0.449
DNase II	0.462
MTBP	0.465
E2F6	2.590
SynCAM	2.359
Ciliated Cell Marker	2.070

CR, Chronic Rejection

### Alteration of STK17A during CR after liver transplantation

To further investigate any clinical consequences for STK17A in CR of liver allografts, immunohistochemistry for STK17A was performed on a total of 52 sequential liver biopsy specimens from 5 pediatric patients with clinically and histologically established CR and their corresponding donors. The clinical and demographic characteristics of these patients are shown in Table 2. The median time from transplantation to the diagnosis of CR and from the diagnosis of CR to the last available follow-up biopsy was 101 days (range 21~401 days) and 1167 days (range 29~1285 days), respectively. Patient 2 lost her graft on postoperative day (POD) 176 due to CR. Most probably, it was a transition from severe ACR to CR. The patient was re-transplanted from her mother, and a follow-up biopsy on POD 2062 showed only mild cholangitis. The remaining 4 patients developed persistent CR, but none developed graft failure at the last available follow-up biopsy. All patients had variable degrees of ACR before the diagnosis of CR. In the early course of CR, liver function was impaired in all patients. Notably, persistent CR was not accompanied by elevation in transaminases, except for alkaline phosphates that remained elevated during the entire course of CR.

**Table 2:** Demographic, clinical characteristics, and IHC findings of serial biopsies of CR cases.

Case	POD	Histological diagnosis	T.Bil (0.3~1.3 mg/dl)	STK17A (BC)	CK7 (BD)
	Original disease [Recipient age at LT; gender], Donor				
1	Fulminant hepatic failure [7 months; male], mother (ABO-C)				
	6	ACR1, Steatosis	1.7	C, D	Normal
	55	ACR2	0.3	C, D	Normal
	72	ACR2	0.5	C, D	Normal
	80	ACR1	0.6	Dis, Fo	Duct atrophy
	101	ICV, Early CR	0.4	Dis, Fo	Duct atrophy
	318	CR, Early	0.5	Dis, Fo	Duct loss, 50%
	430	CR, Early	0.3	Dis, Fo	Duct loss, 30%
	559	CR, Early	0.5	Dis, Fo	Duct loss, 25%
	Follow-up: No graft loss at POD 941				
2	Biliary atresia [12 months; female], father (ABO-I)				
	9	ACR0, Cholestasis	4.6	C, D	Normal
	15	ACR2, Cholestasis	8.6	Dis, Fo	Duct damage
	21	CR, Late	17.3	C, Fo	Duct loss, 67%
	27	CR, Late	19.9	Dis, Fo	Duct loss, 60%
	36	CR, Steatosis	12.5	Dis, Fo	Duct loss, 80%
	50	CR, Persistent	17.4	Dis, Fo	Duct loss, 75%
	Follow-up: Graft failure at POD 176 due to persistent CR				
3	Hepatoblastoma [43 months; male], mother (ABO-C)				
	41	ACR1, Steatosis 10%	4.6	C, D	Ductular reaction
	47	ACR2, Cholangitis	5.4	C, D	Ductular reaction
	54	ACR2, Early CR	9.6	Dis, Fo	Duct loss, 30%
	70	CR, Late	12.6	Dis, Fo	Duct loss, 80%
	96	CR, Late	6	Dis, Fo	Duct loss, 70%
	119	CR, Persistent	3.4	Dis, Fo	Duct loss, 50%
	166	CR, Late	3.5	Dis, Fo	Duct loss, 88%
	371	CR, Late	1.9	Dis, Fo	Duct loss, 55%
	421	Bile duct atrophy	2.9	Dis, Fo	Duct loss, 33%
	513	CR, Late	5.5	Dis, Fo	Duct loss, 67%
	636	CR, Late	2	C, Fo	Duct loss, 90%
	741	CR, Late	1.2	C, Fo	Duct loss, 67%
	869	Cholangitis, CR	2.6	Dis, Fo	Duct loss, 50%
	960	s/o CR	2.1	Dis, Fo	Duct loss, 72%
	1070	CR, late	1.2	Dis, Fo	Duct loss, 62%
	1175	CR, Late	0.7	Dis, D	Duct loss, 60%
	1259	CR, Late	0.7	Dis, Fo	Duct loss, 50%
	1339	CR, Late	0.9	C, Fo	Duct loss, 80%
	Follow-up: No graft loss at POD 1637				
4	Biliary atresia [11 months; female], father (ABO-C)				
	12	ACR0	1.2	C, D	Normal
	76	ACR1	0.3	Dis, Fo	Normal
	92	ACR1	0.7	Dis, Fo	Normal
	159	ACR0, EBV+	1.5	Dis, Fo	Normal
	204	ACR1, Cholestasis	0.1	Dis, Fo	Normal
	395	Lobular inflammation	6.1	Dis, Fo	Duct damage
	401	s/o CR	5.8	C, Fo	Duct loss, 20%
	435	CR, Early	1.7	Dis, Fo	Duct loss, 28%
	708	CR, Late	0.8	C, Fo	Duct loss, 78%
	1066	CR, Late	0.6	C, Fo	Duct loss, 69%
	1591	CR, Early	0.5	Dis, D	Duct loss, 40%
	Follow-up: No graft loss at POD 1591				
5	Tyrosinemia [2 months; male], father (ABO-C)				
	38	ACR1	1.6	C, D	Normal
	68	ACR2, EBV+	1	Dis, D	Normal
	80	ACR, Resolving	0.6	Dis, D	Normal
	138	Portal inflammation	0.6	Dis, Fo	Normal
	292	s/o CR	0.5	Dis, Fo	Duct loss, 78%
	431	s/o CR	0.6	C,Fo	Duct loss, 10%
	795	CR, Early	0.4	C,Fo	Duct loss, 50%
	1145	CR, Early	0.5	C,Fo	Duct loss, 20%
	1459	CR, persistent	0.4	C, D	Duct loss, 25%
	Follow-up: No graft loss at POD 1459				

Abbreviations; ABO-C, ABO-compatible/identical; ABO-I, ABO-incompatible; ACR, acute cellular rejection (0; indeterminate, 1; mild, 2; moderate, 3; severe); BD, bile duct; BC, bile canaliculi; C, continuous; CR, chronic rejection; D, diffuse; Dis, discontinuous; EBV, Epstein–Barr virus; Fo, focal; IHC, immunohistochemistry; ICV, isolated central venulitis; LT, liver transplantation; POD, postoperative day; s/o, suspicious of; T.Bil, total bilirubin.

**Fig 5:**
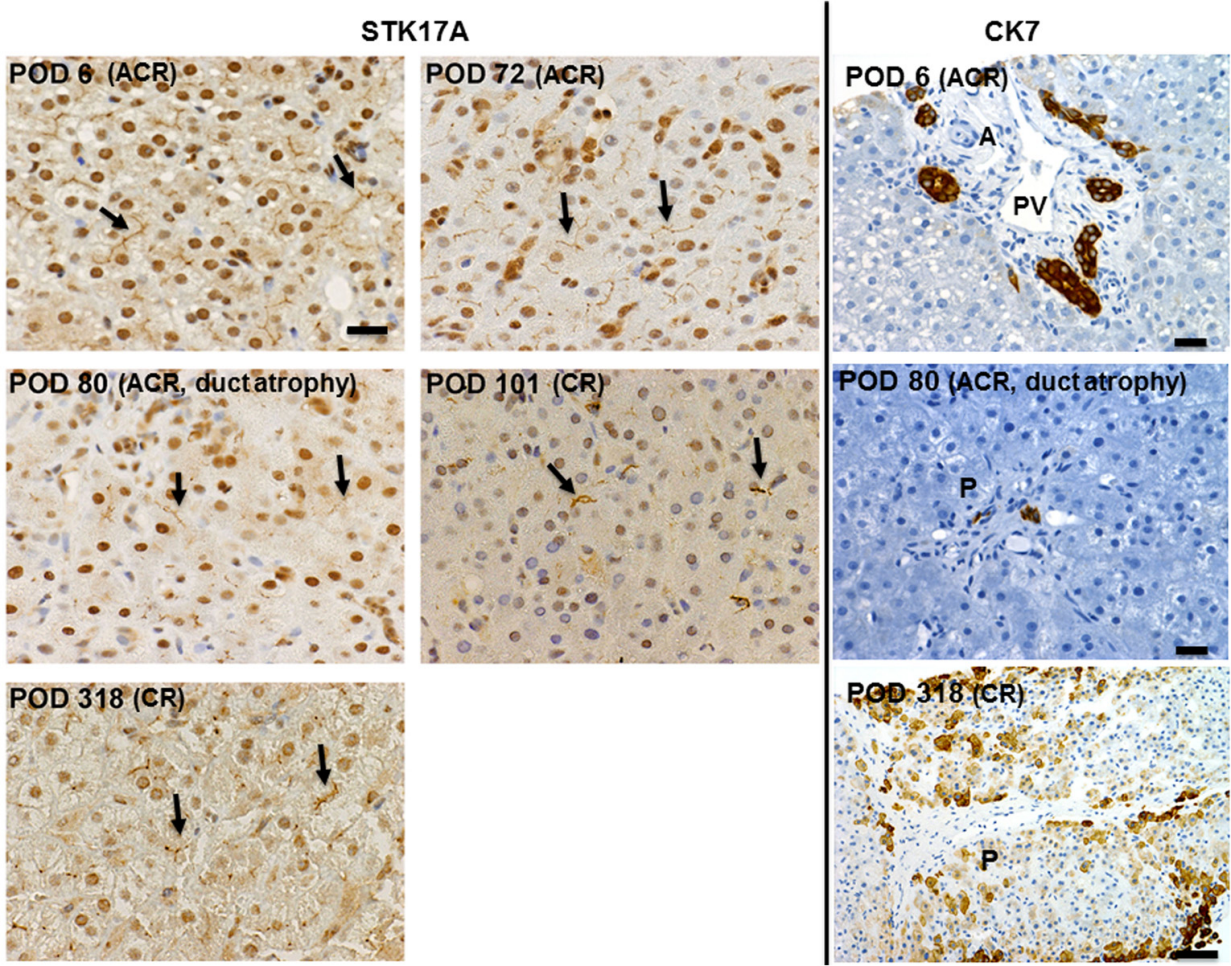
CK7 staining for interlobular bile duct (right panels) and bile canaliculi STK17A staining (left panels) of serial biopsy specimens in patient 1. STK17A staining. (Postoperative day (POD) 6 and 72) Linear, continuous, and diffuse staining pattern. (POD 80, 101, and 318) Discontinuous and focal staining pattern. Scale bar: 20 μm. Arrows in figures point to representative bile canaliculi. CK7 staining. (POD 6) At the time of acute cellular rejection (ACR) showing interlobular bile ducts with well-defined lumen lined by cuboidal epithelium, parallel to an arteriole. (POD 80) In addition to the diagnosis of mild ACR, early CR was suspected due to focal atrophic bile ducts with no well-defined lumen. A = arteriole, PV = portal vein, P = portal area. Scale bars: 20 μm. (POD 318) Absence of bile ducts in portal tract with periportal hepatocyte staining. Scale bar: 50 μm.

For CK7 staining (Fig 5, right panels), the interlobular bile ducts in normal condition are characterized by an intraportal location, parallel to an arteriole, with a well-defined lumen, and lined by cuboidal epithelium. There were atrophic bile ducts with no well-defined lumen at POD 80 and POD 101 in patient 1, for which a definite diagnosis of CR could not be made. A definite diagnosis of CR was made when >50% of the portal area was lacking an identifiable interlobular bile duct (no CK7 staining within the portal area). At CR, CK 7 highlights numerous stained periportal hepatocytes, which may reflect ductopenia [26].

For STK17A immunohistochemistry (Fig 5, left panels), the staining pattern on bile canaliculi was at first solid, linear, and continuous, but became faint and split as CR developed. The area of STK17A staining was changed from diffuse to focal (Table 2). This change was observed before clinical and histological signs of CR and even before detectable bile duct loss on CK7 staining. A discontinuous, split, and focal staining pattern persisted throughout the course of CR. This was accompanied by noticeable bile duct loss. We believe that an alteration in STK17A immunohistochemical staining during the evolution of CR reflects bile canaliculi morphological changes. Intermittent and faint staining was accompanied by canaliculi widening and damage.

### Diffuse staining of STK17A in resolved ACR

**Fig 6:**
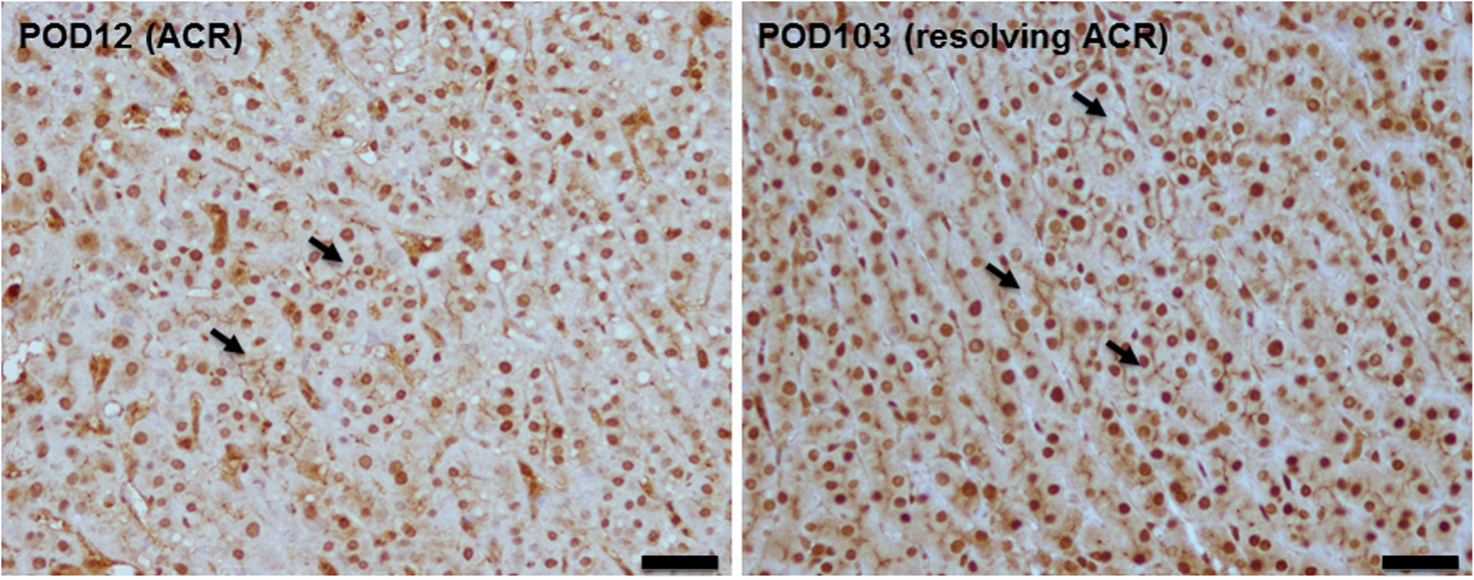
STK17A staining during ACR and at the time of resolution of ACR. Representative figures showing linear, continuous, and diffuse STK17A staining seen at the time of ACR (POD 12) and at the time when ACR was resolved after treatment (POD 103). Scale bars: 20 μm.

Since CR usually evolves from an episode of ACR that is resistant to immunosuppression therapy, it is possible that the ACR process may have triggered the abnormal staining pattern of STK17A in CR. The biopsy time of resolved ACR cases ranged from POD 10~1112, with a median of 87 days. All patients were transplanted due to biliary atresia and received ABO-compatible/identical grafts. Eight of the nine biopsies showed diffuse linear staining. There was no significant difference in the STK17A staining range before and after resolution of ACR (Fig 6); however, there were variable degrees of bile canaliculi widening and damage after ACR. These changes were less than that observed during the course of CR.

### BSEP immunohistochemistry

**Fig 7:**
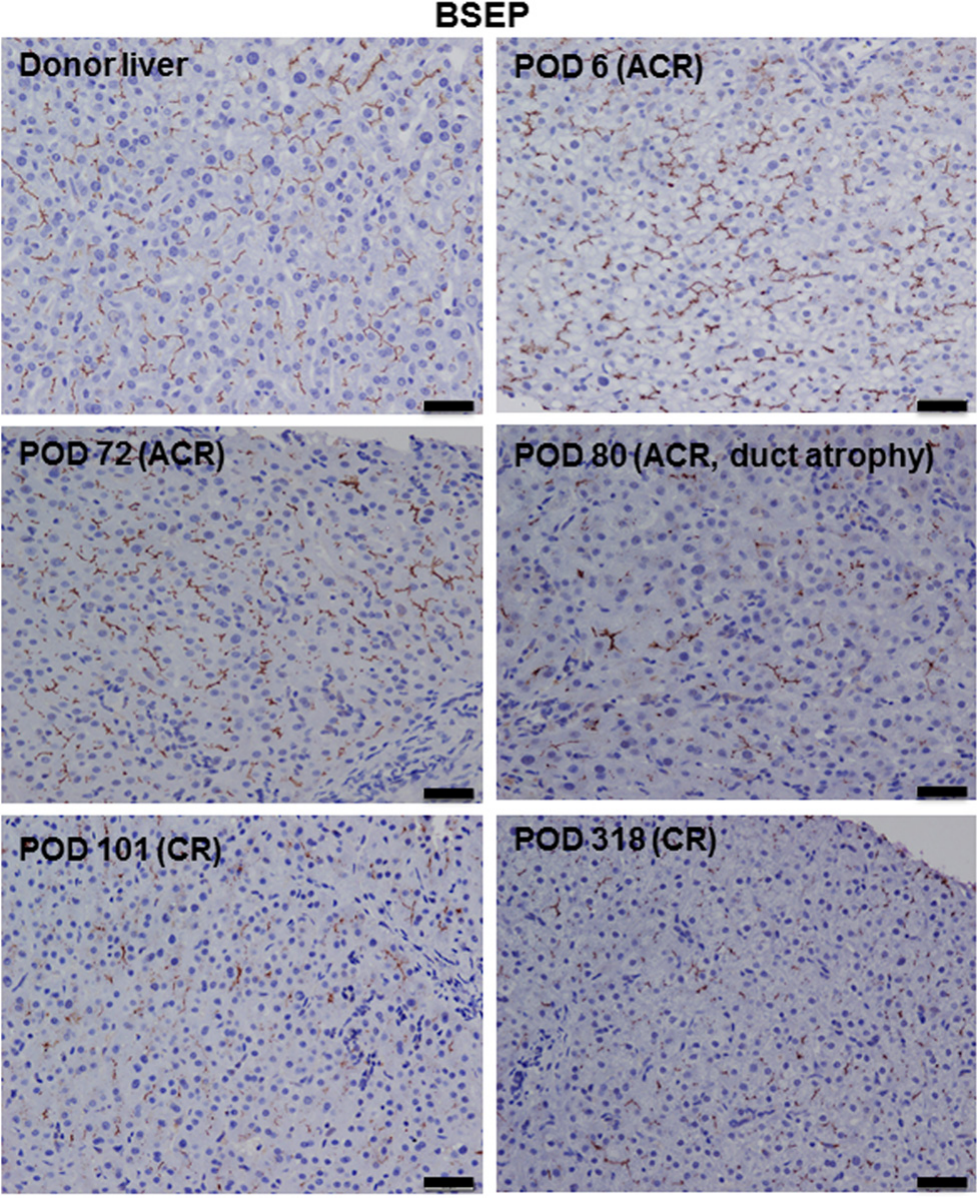
Bile canaliculi BSEP staining in serial biopsies during the course of CR from patient 1. (Donor liver, POD 6, and POD 72) Linear, continuous, and diffuse staining. (POD 80, 101, and 318) Discontinuous and focal staining. Scale bars: 20 μm.

For comparison between traditional bile canalicular markers and STK17A staining, we stained normal livers, failed allografts due to CR, and serial biopsies during CR with BSEP. The staining patterns were nearly similar to STK17A, except no nuclear staining in BSEP (Fig 7).

## Discussion

In the present study, we found a new short isoform of *STK17A* in HepG2 cells. This isoform lacks 34 amino acids. According to the amino acid sequence alignment in DAPK/myosin light-chain kinase (MLCK)/triple functional domain protein (TRIO) family kinases, these 34 amino acids compose segment VIII in the catalytic domain, which is highly conserved in the DAPK family [2,27]. D245 is speculated to be a key amino acid anchoring the regulatory spine. The defective fragment in the *STK17A* short isoform contains the responsible sequence for regulating the conformational change for forming the enzyme-substrate complex in the TRIO family [1]. Indeed, the structural difference between the full-length and the short isoform was predicted by I-TASSER, as shown in Fig 3. Therefore, the short isoform is considered to have an irregular protein function.

This is the first evidence that STK17A localizes at the bile canaliculi in the liver. A group of bile salt export pumps such as MDR, BSEP, MRP, etc. is well characterized to locate bile canaliculi [28]. The functional significance of localization of STK17A in bile canaliculi is obscure. STK17A overexpression induces morphological changes similar to apoptosis in NIH3T3 cells, indicating a role for STK17A in apoptotic signaling [2]. However, the role of STK17A in regulating apoptosis remains controversial. *STK17A* is found to be a novel p53 target gene, and a modulator of cisplatin toxicity and reactive oxygen species in testicular cancer cells [29]. On the other hand, up-regulated *STK17A* in glioblastomas has been reported, where it was associated with tumor grade and patient survival [30]. Moreover, *STK17A* has been found to be overexpressed in human head and neck squamous cell carcinoma by inhibiting the tumor suppressive activity of transforming growth factor (TGF)-beta signaling [31].

A recent report shows that death-associated protein kinase 3 (DAPK3), Rho-associated protein kinase (ROCK), and DRAK kinase regulate the activity of non-muscle myosin II (NM II), which is essential in the regulation of cell signaling, cell adhesion, and tissue structure through phosphorylation of the regulatory light chain (RLC) [7,32]. Neubueser et al. confirmed the role of DRAK in *Drosophila*, in which DRAK was found to stimulate proper morphogenesis of epithelial cells [7]. Based on previous findings and considering normal physiology, in which intracellular actin and myosin filaments surrounding the bile canaliculi drive secreted biliary fluid along the canaliculi into the terminal branches of the bile duct system, STK17A may be involved in maintaining bile canaliculi structure and functionality, probably through phosphorylation of the RLC. Therefore, the reduced expression of STK17A may decrease the assembly of NM II filaments, resulting in impairment of the actin cytoskeleton in bile canaliculi [7,32].

CR is a potentially reversible cause of long-term graft loss [8–13]. However, early diagnosis of CR is still challenging. Our results of Western blotting and immunohistochemistry demonstrated that STK17A expression in CR decreased significantly compared to that in donor livers, which was consistent with the antibody microarray study. The trend of STK17A protein expression on serial biopsies following CR decreased in bile canaliculi during the progression of bile canaliculi destruction, as detected by immunohistochemistry. It is likely that the lower expression of STK17A in the failed allografts due to CR than in the donor livers may reflect the progression of bile canaliculi destruction seen in serial biopsies of CR cases. Moreover, we compared the STK17A immunohistochemical staining pattern in ACR that progressed to CR with ACR that resolved, and found considerably higher damage to bile canaliculi in ACR that progressed to CR.

The etiology of bile canaliculi destruction is not clear; however, direct immune-mediated damage and ischemic damage as a complication of rejection-related vascular injury have been suggested [10,19,33,34]. Loss of the portal tract hepatic artery branches and microcirculation has been found to occur early in CR and to precede bile duct loss [10,33,34]. Additionally, dysplastic bile ducts seen in early CR are associated with overexpression of the senescence-related protein p21 (WAF1/Cip1), which indicates loss of replicative ability and could account for subsequent bile duct loss [10,19].

Since duct loss persisted with the association of the use of regular anti-cellular rejection therapy, the human leukocyte antigen (HLA) donor specific antibody (DSA) test was performed for considering antibody-mediated pathogenesis. The test was positive in case 3 (HLA class I-, II + DSA: DR8 = 2209, DQ4 = 21087; POD, 896) and negative in the other 3 cases. Depiction of any statistical relationship is not possible.

In summary, the localization of STK17A in bile canaliculi is a novel finding. Sequential STK17A immunohistochemical changes seen in bile canaliculi during the development of CR suggest a role for STK17A in conserving bile canaliculi morphology. Assessment of STK17A staining in bile canaliculi could be useful to identify liver recipients who are susceptible to CR. Overall, our work addresses the role of bile canaliculi in maintaining a well-functioning liver allograft and provides insight into the pathogenesis of CR after liver transplantation. Validation of our findings would help in identifying possible molecular mechanisms of CR.
